# Gate modulation of barrier height of unipolar vertically stacked monolayer ReS_2_/MoS_2_ heterojunction

**DOI:** 10.1038/s41598-024-72448-2

**Published:** 2024-09-13

**Authors:** Gowtham Polumati, Chandra Sekhar Reddy Kolli, Aayush Kumar, Mario Flores Salazar, Andres De Luna Bugallo, Parikshit Sahatiya

**Affiliations:** 1 Department of Electrical and Electronics Engineering, BITS Pilani Hyderabad Campus, Hyderabad, 500078 India; 2 Universidad Nacional Autónoma de México, A.P. 1-1010, Querétaro, QRO C.P. 76000 México; 3 Materials Center for Sustainable Energy and Environment, Birla Institute of Technology and Science Pilani, Hyderabad Campus, Hyderabad, 500078 India

**Keywords:** ReS_2_, MoS_2_, vDW heterojunction, FET, Two-dimensional materials, Electronic devices

## Abstract

This study investigates vertically stacked CVD grown ReS_2_/MoS_2_ unipolar heterostructure device as Field Effect Transistor (FET) device wherein ReS_2_ on top acts as drain and MoS_2_ at bottom acts as source. The electrical measurements of ReS_2_/MoS_2_ FET device were carried out and variation in Ids (drain current) Vs Vds (drain voltage) for different Vgs (gate voltage) revealing the n-type device characteristics. Furthermore, the threshold voltage was calculated at the gate bias voltage corresponding to maximum transconductance (g_m_) value which is ~ 12 V. The mobility of the proposed ReS_2_/MoS_2_ heterojunction FET device was calculated as 60.97 cm^2^ V^−1^ s^−1^. The band structure of the fabricated vDW heterostructure was extracted utilizing ultraviolet photoelectron spectroscopy and the UV–visible spectroscopy revealing the formation of 2D electron gas (2DEG) at the ReS_2_/MoS_2_ interface which explains the high carrier mobility of the fabricated FET. The field effect behavior is studied by the modulation of the barrier height across heterojunction and detailed explanation is presented in terms of the charge transport across the heterojunction.

## Introduction

The heterostructure based semiconductor material systems are most preferred in current research in having control over the device charge flow. The proper selection of respective materials and their work functions involved in making heterostructure with its extracted band alignment allows to control carrier migration over the device^1^. Thus, the electronic properties of two different materials involved in heterostructures system can be tailored to make devices such as LEDs, and FETs work suitable for various electronic applications in different fields^2–5^. Transition Metal Dichalcogenides (TMD’s), a two-dimensional semiconductor material have attained significant attention due to their interesting electronic properties making them suitable in next generation FET’s. Among existing TMDs, Molybdenum disulfide (MoS_2_) and Rhenium disulfide (ReS_2_) have attracted exceptional attention because of their remarkable merits in making both FET’s and optoelectronics devices^6–11^.

Monolayer ReS_2_ material with bandgap almost independent from the number of layers with an intrinsic in-plane anisotropy^12–15^ exhibits different electrical and optical properties^16^. This anisotropy can be leveraged to engineer FETs with distinct electronic behavior enabling the creation of FETs that are more sensitive to charge carrier movement in one direction than another^17^. This unique property allows for the design of FETs tailored for specific applications where directional sensitivity is critical. MoS_2_, which is the most studied TMD, has been used in several works in combination with p-type materials^18^. Monolayer MoS_2_ which has a layered dependent bandgap and is exceptionally thin, making it an excellent choice for compact FETs^19^. Its two-dimensional nature promotes efficient charge transport, leading to high-speed electronic devices^6,20–23^. Additionally, the scalability and versatility of MoS_2_ make it an attractive material for FETs that need to be integrated into diverse electronic systems^24^.

The proposed heterostructure FET device which has vertically stacked configuration (ReS_2_ on top) taking ReS_2_ as drain and MoS_2_ as source will result in type-1 band alignment upon heterostructure formation^25^. The type-1 band alignment in heterostructure FET devices will have many advantages when compared to pristine FET counter parts such as efficient electron and hole transfer, low resistance at the interface (channel), reduced scattering, enhanced carrier transport and optimized charge injection. which makes the heterostructure FET device promising in many high-speed device applications^26^.

The proposed ReS_2_/MoS_2_ heterostructure device will have a combined properties of ReS_2_ and MoS_2_ such as efficient charge migration, reduced recombination, control over threshold voltage and highspeed operation at low energy consumption making the device suitable in many practical applications demanding high speed switching operations with appreciable sensitivity^27^. The ReS_2_/MoS_2_ (ReS_2_ on top) heterostructure FET device was grown on p-type Si/SiO_2_ substrate using 2 step CVD method. Accordingly, MoS_2_, ReS_2_ and p-type Si/SiO_2_ were taken as source, drain and gate accesses regions respectively with the interface between ReS_2_ and MoS_2_ acting as channel. The as grown ReS_2_/MoS_2_ heterostructure will have a type-1 band alignment supporting efficient transport of charge carriers from source to drain upon applied voltage. Electrical measurements of ReS_2_/MoS_2_ FET device were carried out and variation in Ids (Drain current) with Vds (Drain voltage) for different Vgs (Gate voltage) were observed. Wherein it was concluded that with increase in voltage Vds the conduction band energy of drain (ReS_2_) will be decreased which lies lower than that of conduction band energy of source (MoS_2_), creating clear path for the charge carriers to migrate from source to drain and producing drain current Ids. The increase in Ids with increase in applied Vds may be attributed to effective migration of charge carriers from source to drain which is due to lowering of conduction band energy of drain (ReS_2_) compared to Source (MoS_2_). Similarly, transfer characteristics (Ids Vs Vgs) for different Vds reveals that the increase in Ids with increase in Vgs was because the voltage Vgs applied across the gate efficiently modulates conduction portion(channel) of the device and has efficient control over the current Ids. Furthermore, the threshold voltage was calculated at the gate bias voltage corresponding to maximum transconductance (gm) value which is ~ 12 V. The mobility of the proposed ReS_2_/MoS_2_ heterostructure FET device was calculated as 60.97 cm^2^ V^−1^ s^−1^. Therefore, it is clearly understood that the exceptional value of mobility is due to formation of type-1 band alignment between MoS_2_ and ReS_2_ heterostructure along with 2DEG at the interface which may considered as major factor in increasing the mobility of the device. The increase in mobility is attributed to decreased impurity scattering of 2DEG at the interface which has added benefits of having high-speed switching due to less recombination making the device suitable in many practical and potential applications in demand.

## Experimental section

### *Synthesis of* ReS_2_/MoS_2_*heterojunctions using CVD*

**Fig. 1:**
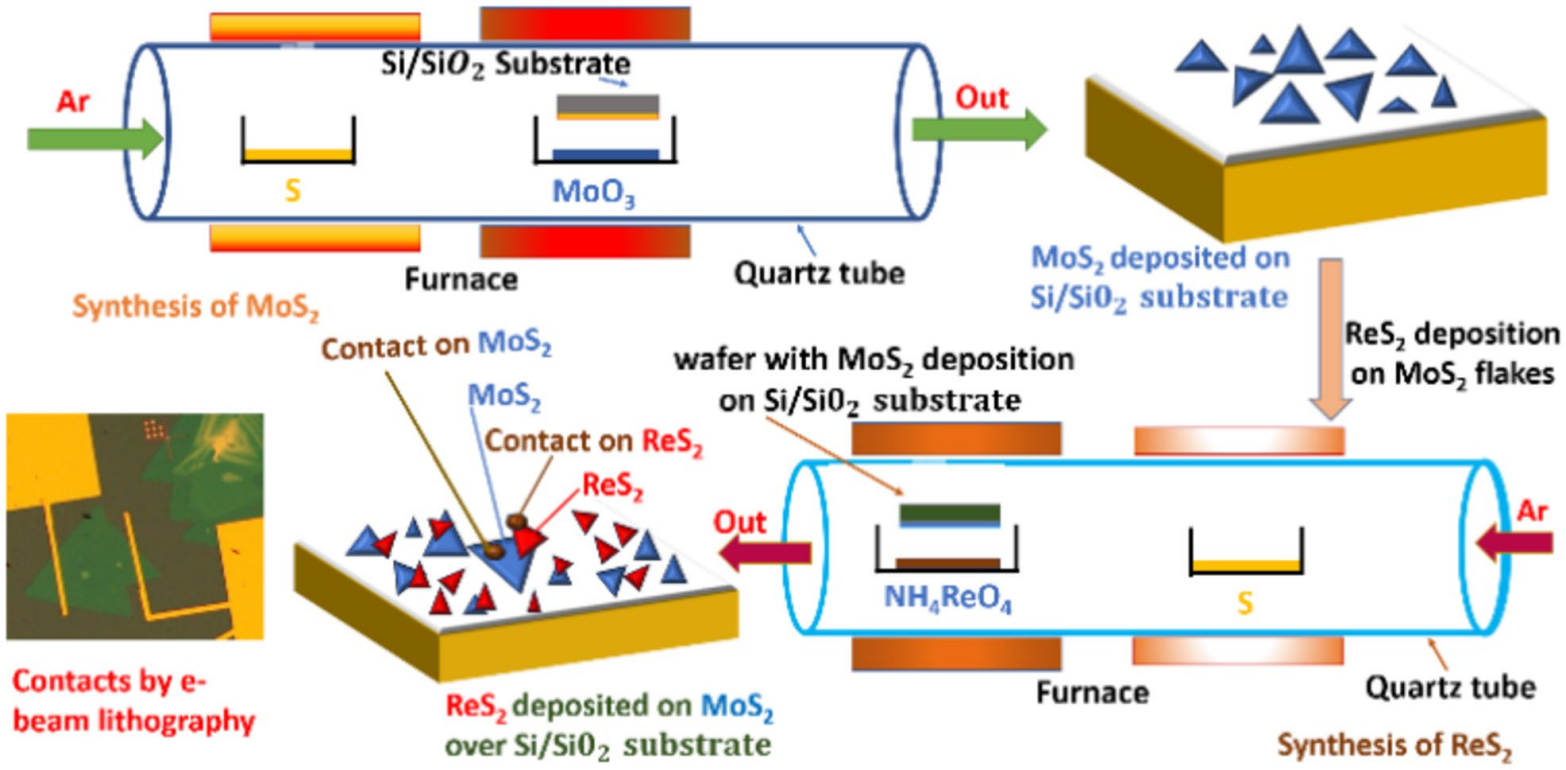
CVD schematic showing the complete synthesis of ReS_2_/MoS_2_ FET device.

The synthesis of vertically stacked ReS_2_/MoS_2_ (ReS_2_ on top) heterostructure device was carried out using 2 step CVD method^5^. In 1st step the growth of monolayer MoS_2_ was carried out by taking molybdenum trioxide (MoO_3_) of 140 mg and sulfur (S) powder of 200 mg in aluminum oxide (Al_2_O_3_) boats as metal and chalcogen precursors. p-type silicon is used as substrate, which a has thickness of 280 nm and doping concentration of 6.25 × 10^17^/cm^3^.The substrate p-type Si/SiO_2_ substrate having dimensions of (1.5 cm × 1.5 cm) was subjected to RCA cleaning and then placed facing down on the boat having MoO_3_ powder. Prior to reaction, the quartz tube was connected to mechanical pump for creating vacuum. Once the vacuum is created, the carrier gas (Ar) was purged inside the quartz tube till it reaches atmospheric pressure. Then after, the reaction temperatures of both MoO_3_ and sulfur powder were set to reach simultaneously and maintained constant at 850 °C and 200 °C respectively for 20 min by keeping gas flow rate at 50 sccm. Once the reaction is done, the quartz tube is left undisturbed to natural cooling. In 2nd step, similar procedure was followed for subsequent deposition of monolayer ReS_2_. The MoS_2_ grown substrate was placed facing down on boat having Re source. 50 mg of Ammonium Perrhenate (NH_4_ReO_4_) and 150 mg of sulfur powder were taken as Re and S precursors. The temperatures of both the zones were maintained constant at 750 °C and 150 °C respectively for 10 min with carrier gas (Ar) flow rate of 50 sccm. Once the deposition of flakes was complete, metallic patterns and contact making was performed employing standard photolithography and E-beam lithography. Thermal evaporation was employed for coating metallization of Ti (10 nm)/Au (200 nm) followed by lift-off procedure to complete device fabrication. Schematic showing detailed device configuration is shown in Fig. 1.

## Results and discussion

**Fig. 2:**
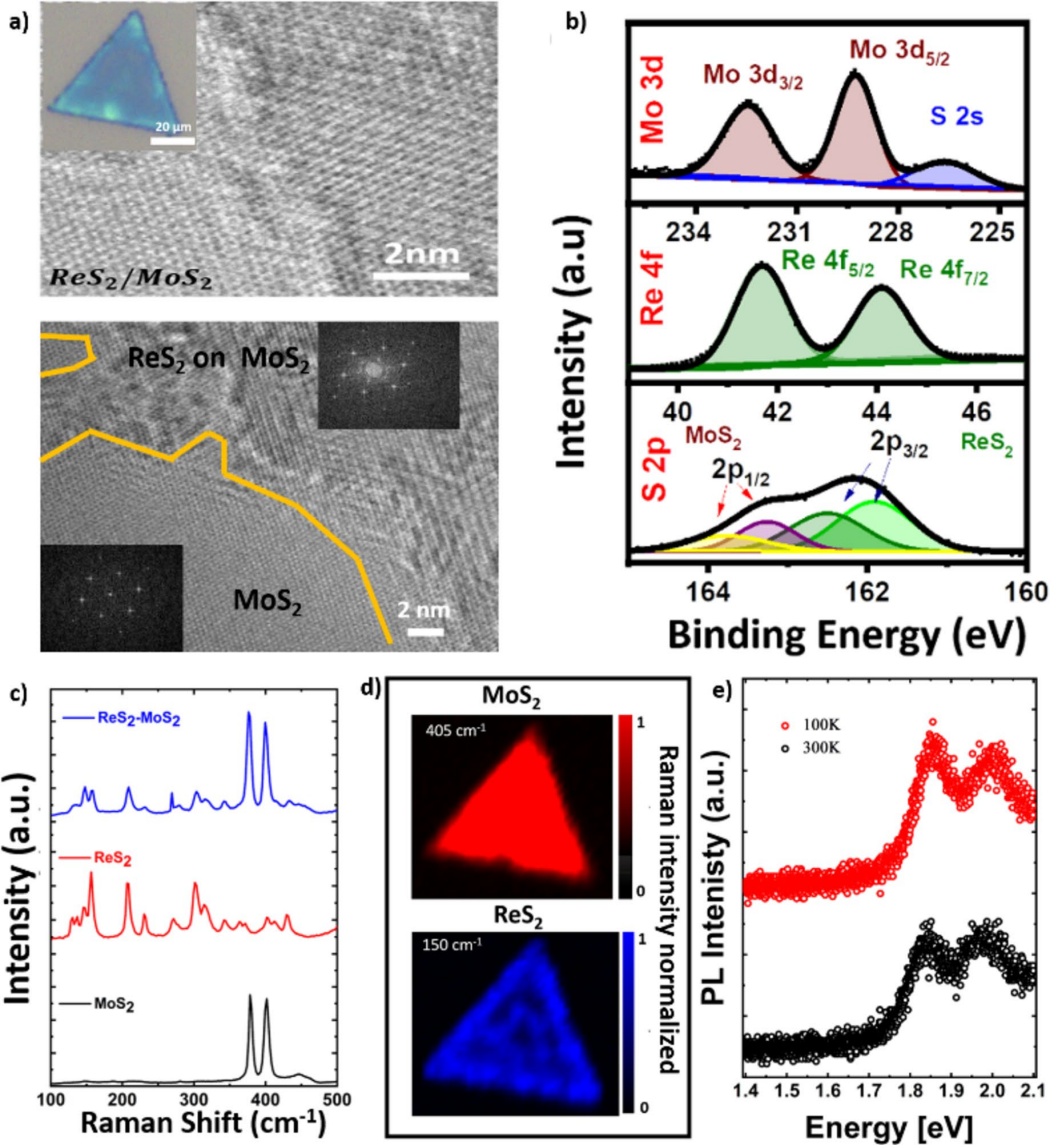
Complete characterization of ReS_2_/MoS_2_ Heterostructure. (**a**) HR-TEM (inset illustrates optical images) of the ReS_2_/MoS_2_ heterostructure after the two-step CVD growth. (**b**) Representative XPS spectra of the ReS_2_/MoS_2_ samples. (**c**) Raman spectra between pristine samples and heterostructures. (**d**) Raman mapping on the heterostructures (**e**) PL of heterostructure.

The formation of ReS_2_/MoS_2_ heterostructure was initially examined by optical images where the overlap of MoS_2_ and ReS_2_ was visually observed as shown in inset of Fig. 2a. To further investigate the structural relation between ReS_2_ and MoS_2_ of the heterostructure, high-resolution transmission electron microscopy (HRTEM) was performed. Figure 2a shows HRTEM and their fast Fourier transform images showing an epitaxial relation between MoS_2_ and ReS_2_ confirming formation of vertical heterostructure**.** wherein it was concluded that the rhenium atoms acquire diamond chain like arrangement which are aligned to principal directions with sulfur atoms being organized to form distorted octahedral structure and thus making ReS_2_ to have triclinic structure (1 T)^28^.Therefore, it was then concluded that heterostructure has triclinic nature of ReS_2_ and hexagonal nature of MoS_2_. Furthermore, to have an intense examination of the as grown heterostructure semiconductor device and its chemical composition, the device was subjected to X-Ray photoelectron spectroscopy (XPS)^29–32^. Figure 2b shows the corresponding spectra of Mo 3d, Re 4f. and S2p of the device^33^. The as observed doublets of S2p i.e. S 2P_1/2_ and S 2P_3/2_ corresponds to ReS_2_ and MoS_2_ indicating that the device is made of ReS_2_/MoS_2_ semiconductor materials. The interaction between both the materials was further investigated by Raman spectroscopy. The upper spectrum reveals E_2_g and A1g modes of MoS_2_ which are located around 384 cm^−1^ and 402 cm^−1^ while the lower spectrum reveals the in-plane (Eg) and out-of-plane (Ag) modes of ReS_2_^34^. It was clearly observed that the Raman spectra of ReS_2_/MoS_2_ heterostructure has a blue shift when compared to bare ReS_2_ (MoS_2_) as can be clearly seen in Fig. 2c. The blue shift in the heterostructure with no other peaks indicates that existence of good interlayer coupling between the ReS_2_ and MoS_2_ materials having both vertical and lateral growth^35^.To examine spatial distribution, Raman mapping was performed on the heterostructures as shown in Fig. 2d. The results indicate that both materials are present throughout the flakes, confirming the presence of vertical stacking**.** The ReS_2_/MoS_2_ heterostructure was further examined using photoluminescence (PL) spectroscopy at both room temperature (300 K) and 100 K. The spectra at both temperatures reveal the presence of exciton A and exciton B, corresponding to the direct transitions between the conduction band minima and the valence band maxima of MoS_2_ at the gamma point of the Brillouin zone. However, the PL intensity is very low, which is attributed to charge transfer from MoS_2_ to ReS_2_ due to the formation of a type I heterostructure alignment^36^. As evident from the PL emission displayed in Fig. 2e, there is a notable quenching in the PL intensity of MoS_2_, with no observable PL peak corresponding to ReS_2_ emission. This quenching is consistent with the findings of previous studies^1,25,37^. Our analysis suggests that the low PL intensity of MoS_2_ is due to charge transfer from MoS_2_ to ReS_2_ due to the formation of a type I heterostructure, which reduces the recombination rate in the MoS_2_ layer. It is also important to noticed that in our experiments we did not observe any PL emission from ReS_2_. We believe this absence of emission is due to several factors: ReS_2_ monolayers typically exhibit low quantum yields, possess a significant effective mass for electrons and carriers, and are inherently n-type semiconductors. The charge transfer from MoS_2_ to ReS_2_ likely increases the electron concentration in the ReS_2_ monolayer, which promotes the formation of charged excitons. This process could result in the observed reduction in PL emission from ReS_2_. However, further experiments are needed to validate these conclusions.

**Fig. 3:**
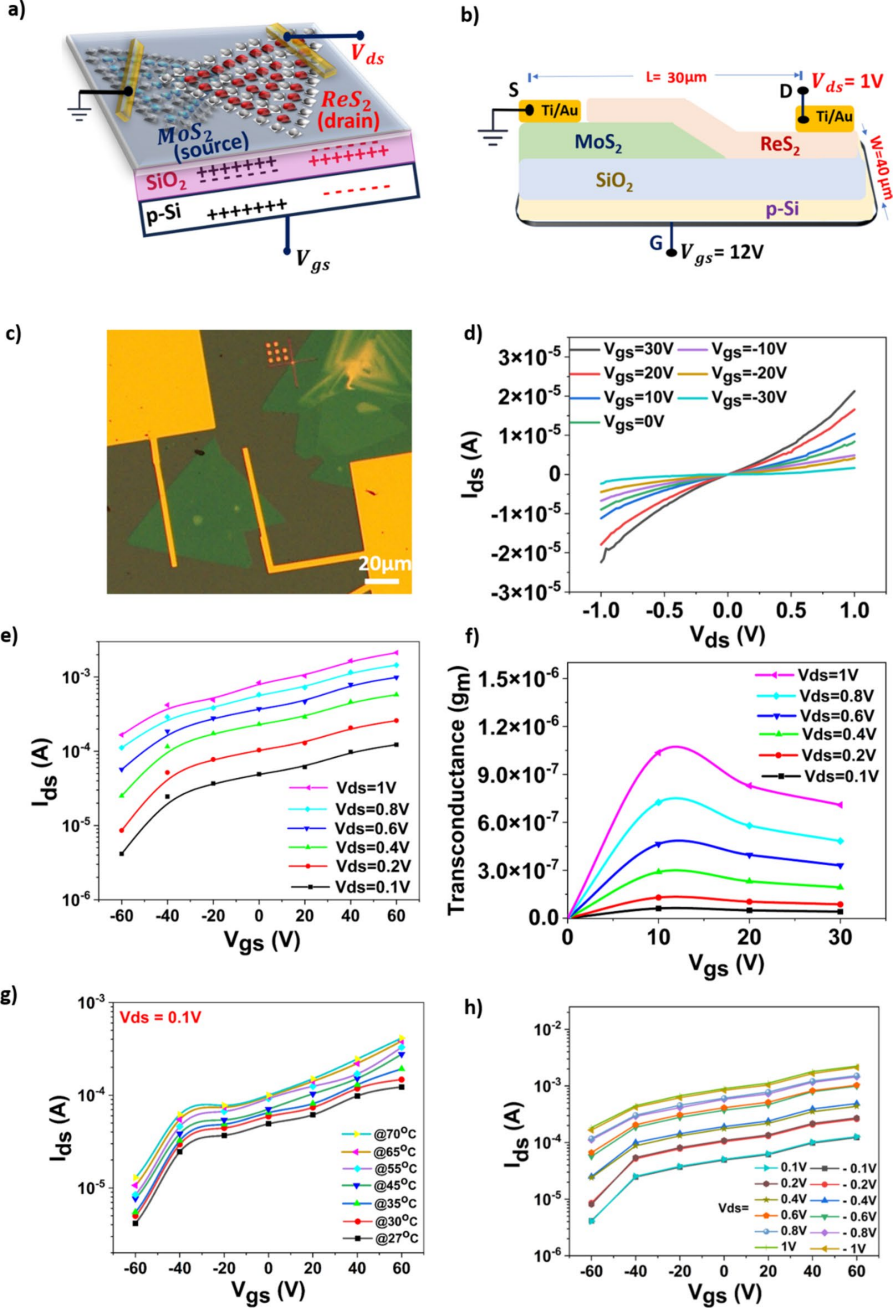
(**a**) Schematic showing the ReS_2_/MoS_2_ FET device. (**b**) Device configuration with bias. (**c**) e-beam image of the as fabricated FET device, (**d**) I-V curves as a function of different Vgs voltages. (**e**) Transfer characteristics of the fabricated ReS_2_/MoS_2_ FET when Vds =  + ve (**f**) Variation of gm v/s Vgs to extract the threshold voltage of the fabricated ReS_2_/MoS_2_ FET. (**g**) Graph showing variation in Ids with increase in device temperature. (**h**) Transfer characteristics of the fabricated ReS_2_/MoS_2_ FET when Vds =  +ve/−ve.

Figure 3a shows the device configuration of ReS_2_/MoS_2_ (ReS_2_ on top) vertically stacked vdws heterostructure FET device of which MoS_2_ acts as source and ReS_2_ as drain which are grown on p-type Si/SiO_2_ substrate acting as back gate with the overlap region between source (MoS_2_) and drain (ReS_2_) as channel. The authors have previously reported the values of concentration of electrons and holes for both MoS_2_ and ReS_2_ wherein the concentration of charge carriers was found to be higher for ReS_2_. Hence ReS_2_ was chosen as the forcing electrode as it would be able to provide more charge carrier when compared to MoS_2_^38^. Also, the ReS_2_ on MoS_2_ heterostructure configuration forms type-I band alignment and MoS_2_ on ReS_2_ yields type II band alignment. It is well known fact that type-1 band alignment will have quantum wells for both electrons and holes which further helps in better or improved conductivity^5^. The source and drain materials are chosen to ensure appreciable charge transfer as such device configuration (ReS_2_ on top of MoS_2_) will give a type-1 band structure providing quantum wells for both electrons and holes^39^. The length and width of the ReS_2_/MoS_2_ FET device were taken as 30 μm and 40 μm respectively as shown in Fig. 3b. Also, Ti (10 nm)/Au (200 nm) contacts which acts as source and drain were taken from vertically stacked MoS_2_ and ReS_2_ respectively as shown in Fig. 3c. Furthermore, the electrical measurements of ReS_2_/MoS_2_ FET device were carried out as shown in Fig. 3d which illustrates Ids (Drain current) Vs Vds (Drain voltage) for different Vgs (Gate voltage). Wherein it was clearly observed that with increase in voltage Vds, current Ids increases and vice -versa, indicating the charge transfer from source to drain (charge transfer happens from drain to source when Vds is made negative). The non-ohmic nature of the device in Fig. 3d is due to work function difference of Ti/Au contact with MoS_2_ and ReS_2_. In general while fabricating FET devices, the metal work function is chosen to be close to electron affinity of either MoS_2_/ReS_2_. However considering the work function of Ti/Au to be 4.33 eV and monolayer MoS_2_ and monolayer ReS_2_ to be 4.28 eV and 4.84 eV respectively. The intrinsic defects present in both MoS_2_ and ReS_2_ results in fermi level pinning resulting in rectifying contacts irrespective of metal contact work function. This phenomenon was frequently reported in previous studies. Furthermore, the prolonged exposure of Ti contact to open atmosphere and unreacted residuals of MoO_3_ (in case of MoS_2_) might result in local oxidation of Ti by diffusion process leading to increase in work function value of TiO_2_ to 4.9–5.5 eV resulting in rectifying experimental observations^40–42^.Similarly, electrical measurements were also carried out for ReS_2_/MoS_2_ FET device to observe the transfer characteristics (Ids Vs Vgs) for different Vds as shown in Fig. 3e**.** The increase in Ids with increase in Vgs was because of the voltage Vgs applied across the gate efficiently improves conduction over the channel there by increasing the current Ids. Furthermore, the threshold voltage was calculated at the gate bias voltage corresponding to maximum transconductance (gm) value which is ~ 12 V as can be seen from gm Vs Vgs plot as in Fig. 3f. The electrical measurements were also carried at Vds = 0.1 V to examine the device behavior with increase in temperature. wherein an increase in the drain current was observed with increase in temperature which attributes to efficient carrier generation upon subjecting the device to temperature as shown in Fig. 3g. The transfer characteristics with hysteresis plot when Vds =  + ve/-ve is examined shown in Fig. 3h.The irregular trend in transfer characteristics are due to nature of heterostructure. It is to note that the transfer characteristics shows the irregular trend in accordance with increase and decrease in Vds. With increase in Vds the current Ids tends to flow from source (MoS_2_) to drain (ReS_2_) and with decrease in Vds it changes its direction and flows from drain (ReS_2_) to source (MoS_2_). As the experiment is conducted with both + Vds and −Vds, there is always an offset current due to carriers inside the channel being tend to change in their direction with change in bias voltage eventually leading to irregular current trend in response to drain voltage Vds.

The carrier mobility (*μ*) of the fabricated ReS_2_/MoS_2_ FET device was calculated using the following equation^43^.

$$\upmu =\frac{{dI}_{DS}}{{dV}_{GS}}\frac{L}{W.{V}_{DS}.Cg}$$

where I_D_ is the drain current, W is the channel width 40 µm, L is the channel length 30 µm, and V_d_ = 1 V is the drain bias, C_g_ = 123 µF is the gate capacitance which is calculated as C_g_ = ε_ox_/t_ox_, where ε_ox_ = 3.9ε_o_ and t_ox_ = 280 nm is the oxide thickness for SiO_2_(commercially available Si/SiO_2_). The mobility value calculated was ≈ 60.97 cm^2^ V^−1^ s^−1^.The device configuration with above parameters and biasing supplies was shown in Fig. 3b. The exceptional mobility of the device is suggested to formation of type-1 band alignment between MoS_2_ and ReS_2_ heterostructure along with 2DEG at the interface.

### ***ReS***_***2***_***/MoS***_***2***_*** FET device mechanism***

The charge migration and carrier transportation of the ReS_2_/MoS_2_ FET device was understood by extracting its band structure using ultraviolet photoelectron spectroscopy (UPS).

**Fig. 4:**
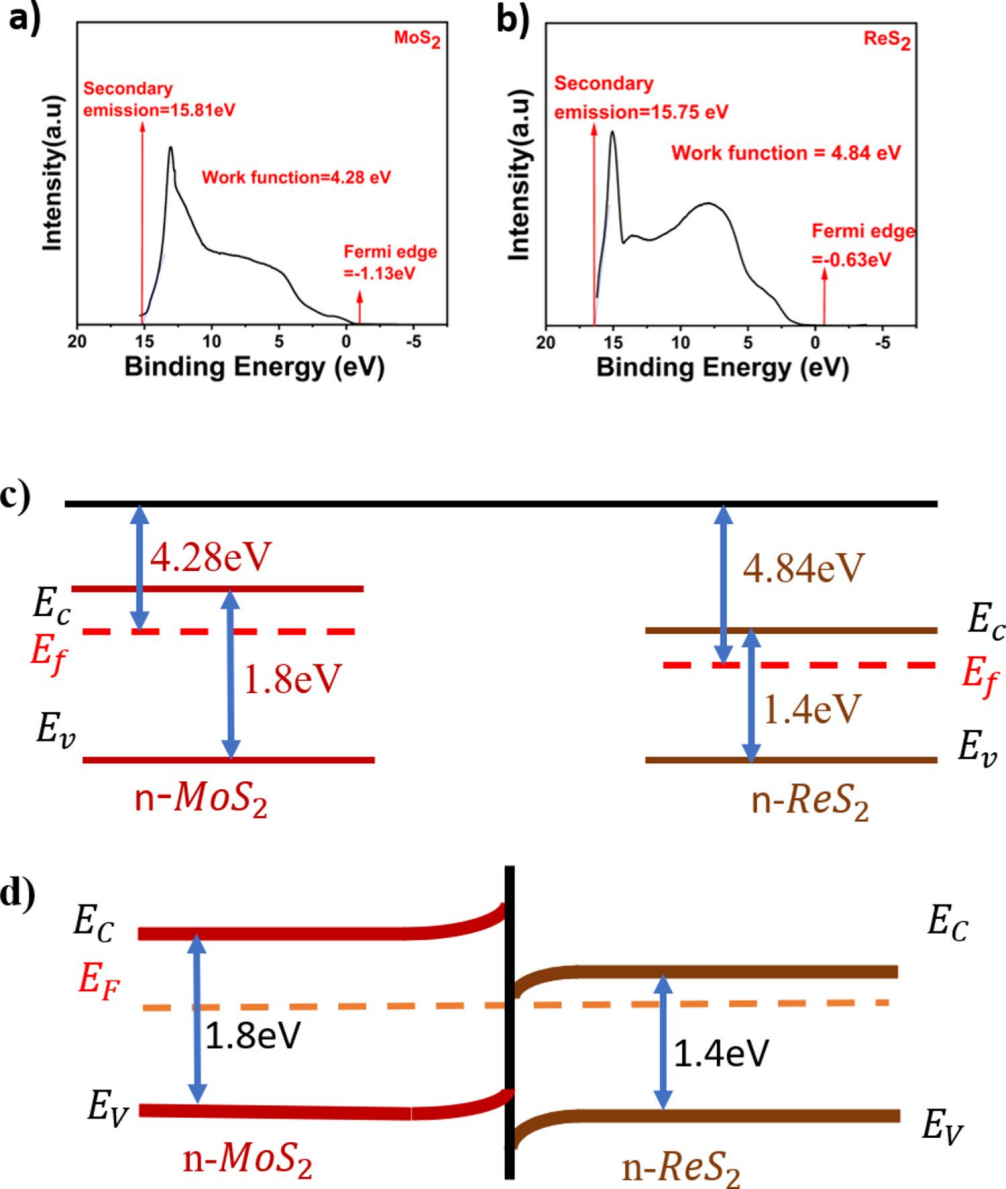
Extraction of band structure using UPS plots. (**a**–**b**) UPS spectra (measured by He I source, hν = 21.22 eV) of pristine MoS_2_ and pristine ReS_2_. (**c**) Energy band diagram of pristine ReS_2_ and MoS_2_ when isolated. (**d**) Energy band diagram ReS_2_ and MoS_2_ when contacted.

The work functions of both ReS_2_ and MoS_2_ are calculated using UPS plots as shown in Fig. 4a,b. The secondary electron cut-off energy of MoS_2_ and ReS_2_ was measured as 15.81 eV and 15.75 eV respectively. The calculated work functions by subtracting the cut off energy from excitation energy (He I, 21.22 eV) was 4.28 eV (MoS_2_) and 4.84 eV (ReS_2_) respectively^38^. The extracted band structures of both MoS_2_ and ReS_2_ when isolated and contacted are shown in Fig. 4c,d. ReS_2_/MoS_2_ FET device is the heterojunction device which has an interface separating ReS_2_ and MoS_2_ semiconductor materials. When both these materials are joined, carrier migration takes place to attain equilibrium which in turn develops internal built-in potential limiting further transfer of charge at the interface^44^. As a result, fermi level is aligned uniform throughout the heterojunction device. Consequently, the band bending and barrier formation takes place at the interface to confine carriers and these confined carriers are major responsible in device current when subjected to external trigger.

**Fig. 5:**
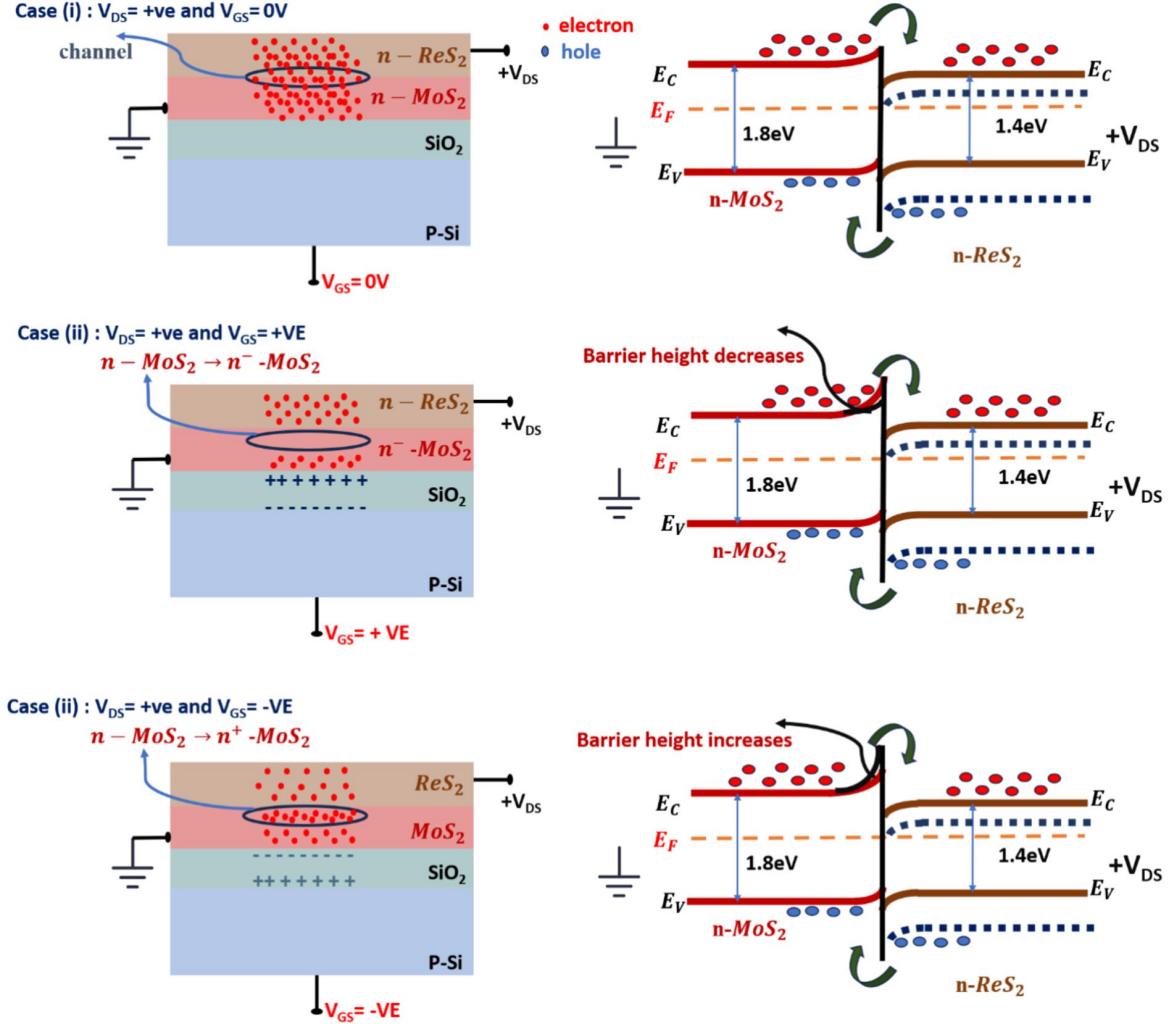
case(i) Variation of Ids with positive Vds when Vgs = 0. Case(ii) Variation of Ids with positive Vgs when Vds =  + ve. Case(iii) Variation of Ids with negative Vgs when Vds =  + ve.

The drain and transfer characteristics of the ReS_2_/MoS_2_ FET device is explained in three different cases as shown in Fig. 5. Considering MoS_2_ as source, ReS_2_ as drain, p-type Si/SiO_2_ as gate and interface of ReS_2_/MoS_2_ acting as channel.

#### Case(i)

Vgs = 0 V, Vds =  + VE

Under no gate voltage (Vgs = 0), when the drain (ReS_2_) is connected to positive and source (MoS_2_) is connected to ground. The conduction band energy of ReS_2_ will be lowered compared to conduction band of MoS_2_ (as shown with dotted lines in case (i) of Fig. 5 thus making path for carriers to travel from source to drain and consequently charge migration happens from source (conduction band of MoS_2_) to drain (conduction band of ReS_2_). Therefore, the drain current Ids increases non-linearly with increase in Vds**.**

#### Case(ii)

Vgs =  + ve, Vds =  + VE

When positive gate voltage is applied, the SiO_2_ layer will be polarized. Consequently, it attracts carriers (electrons) from the interface (channel) making n-type MoS_2_ to n^−^MoS_2_ (less n type) at the interface as shown in case (ii) of Fig. 5. Thus, the band structure of n-MoS_2_ will change at the interface corresponding to n^−^-MoS_2_. As result, the barrier height of MoS_2_ at the interface will decrease and the decrease in barrier height will be in accordance with amount of gate voltage applied and grade of SiO_2_ polarization. Due to decrease in barrier height at the interface, the charge carriers which are to migrate from conduction band of source (MoS_2_) to conduction band of drain (ReS_2_) will have feasible access in crossing the interface with negligible recombination’s to reach the drain terminal. As a result, the ReS_2_/MoS_2_ FET device shows increase in drain current with increase in applied positive gate voltage.

#### Case(iii)

Vgs = −ve, Vds =  + VE

Similar study was done for negative gate voltage, when negative gate voltage is applied, the SiO_2_ layer will be polarized and accordingly the carriers(electrons) are being repelled towards interface converting n-type MoS_2_ to n^+^-MoS_2_ at the interface(channel) as shown in case (iii) of Fig. 5. As a result, the band structure of n-type MoS_2_ at the interface will change corresponding to n^+^-MoS_2_. Thus, the barrier height of MoS_2_ at the interface will increase and the increase in barrier height is in accordance with applied gate voltage and the degree of SiO_2_ polarization. Due to increase in barrier height at the interface, the charge carriers which are supposed to migrate from conduction band of source (MoS_2_) to conduction band of drain (ReS_2_) will have difficult access to cross the interface and might recombine resulting in decrease in overall drain current. As a result, the ReS_2_/MoS_2_ FET device shows decrease in drain current when negative gate voltage is applied. It should be noted that, when Vds < 0, the device current tends to decrease for both case (ii) & case (iii) as in such case current tend to change in its direction from source to drain (instead of from drain to source). As in such case, carriers will experience considerable barrier height resulting in decreased current.

Sebastian et al. have fabricated FETs using pristine monolayer WS_2_ and MoS_2_ using metal organic CVD and noted mobility of the device as 33 cm^2^ V^−1^ s^−1^ and 23.9 cm^2^ V^−1^ s^−1^. Shin et al. have fabricated a vertically stacked FET using graphene and WSe_2_ and measured mobility 0.000012 cm^2^ V^−1^ s^−1^. Zhang et al*.* also developed a FET using pristine ReS_2_ using CVD technique calculated carrier mobility 1 cm^2^ V^−1^ s^−1^. Qu et al. have fabricated a FET using multilayer MoS_2_ and graphene by mechanical exfoliation and measures mobility 170 cm^2^ V^−1^ s^−1^. Zou et al*.* fabricated three devices using monolayer MoS_2_, 4 nm thick MoS_2_, and 6 nm thick MoS_2_ and calculated carrier mobility (electron mobility) for as 30 cm^2^ V^−1^ s^−1^, 1.1 cm^2^ V^−1^ s^−1^ and 3 cm^2^ V^−1^ s^−1^respectively*. *Fang et al. developed a field effect transistor based on bilayer MoS_2_ and measured electron (carrier) mobility as 45 cm^2^ V^−1^ s^−1^. Kaushik et al. have fabricated FET using MoS_2_/WS_2_ heterostructure and calculated carrier mobility as 19 cm^2^ V^−1^ s^−1^ and that for pristine MoS_2_ FET and WS_2_ FET the measured mobilities are 1.23 cm^2^ V^−1^ s^−1^ and 3.32 cm^2^ V^−1^ s^−1^. Shin et al. performed experiment GaS − MoS_2_ van der Waals Heterostructure Based Field-Effect Transistor and calculated mobility as 83 cm^2^ V^−1^ s^−1^. Kim et al. have fabricated high-mobility junction FET transistor via graphene/MoS_2_ heterointerface and calculated mobility as 100 cm^2^ V^−1^ s^−1^. Lee et al. have conducted experiment on modulation doping in van der Waals heterostructure transistors using InSe/hBN/graphite interface and noted mobility as 60 cm^2^ V^−1^ s^−1^. Wu et al. developed InSe/hBN/graphite heterostructure FET for high-performance 2D electronics and noted mobility as 1146 cm^2^ V^−1^ s^−1^. Intonti et al. and Durante et al. have developed ReS_2_ based FETs and examined mobilities as 3 cm^2^ V^−1^ s^−1^ and 6 cm^2^ V^−1^ s^−1^ respectively.

The proposed ReS_2_/MoS_2_ heterostructure FET device in this work delivers carrier mobility of 60.97 cm^2^ V^−1^ s^−1^ which is exceptionally good compared to many devices which are fabricated using different synthesis procedures stated below in Table 1. It was concluded that the device delivers appreciable mobility due to type-1 band alignment between MoS_2_ and ReS_2_ interface of the heterostructure. And it was a proven fact that the type-1 band alignment supports many added advantages in charge transport mechanism with negligible recombination’s in heterostructure FET devices.

**Table 1:** Comparision of various device configurations with proposed device.

Device material	Synthesis procedure	Substrate	Mobility (cm^2^ V^−1^ s^−1^)	References
WS_2_	MOCVD	Si/SiO_2_	33	^36^
MoS_2_	MOCVD	Si/SiO_2_	23.9	
WSe_2_-graphene	CVD	Si/SiO_2_	0.000012	^45^
ReS_2_	CVD	Si/SiO_2_	1	^46^
MoS_2_–graphene	Exfoliation	Si/SiO_2_	170	^44^
MoS_2_	Exfoliation	Si/SiO_2_	0.22	^47^
MoS_2_	Exfoliation	Si/SiO_2_	30	^48^
MoS_2_	Exfoliation	Si/SiO_2_	1.1	^48^
MoS_2_	Exfoliation	Si/SiO_2_	3	^48^
Bilayer MoS_2_	CVD	Si/SiO_2_	45	^49^
MoS_2_-WS_2_	CVD	Si/SiO_2_	19	^50^
MoS_2_	CVD	Si/SiO_2_	1.23	^50^
WS_2_	CVD	Si/SiO_2_	3.32	^50^
GaS/MoS2	CVD	Si/SiO_2_	83	^51^
Graphene/MoS2	CVD	Si/SiO_2_	100	^49^
Wse2/MoS2	CVD	Si/SiO_2_	60	^50^
ReS_2_	Exfoliation	Si/SiO_2_	3	^52^
ReS_2_	Exfoliation	Si/SiO_2_	6	^53^
BP/MoS_2_	CVD	Si/SiO_2_	–	^18^
InSe/hBN/graphite	Exfoliation	Si/SiO_2_	1146	^54^
ReS_2_/MoS_2_	CVD	Si/SiO_2_	60.97	This work

## Conclusion

In conclusion, this work demonstrates the fabrication of ReS_2_/MoS_2_ FET device which was grown on p-type Si/Sio_2_ substrate using 2 step CVD method. It was observed that the as grown heterostructure resulted in type-1 band alignment promoting efficient charge migration from source to drain. Furthermore, the ReS_2_/MoS_2_ FET device was subjected to various electrical measurements wherein it was observed that the conduction portion(channel) of the device is efficiently controlled by gate terminal. The drain and transfer characteristics of the proposed device were extracted and observed significant variation of Ids with Vds(Vgs). Accordingly, the mobility of the proposed ReS_2_/MoS_2_ heterostructure FET device was calculated as 60.97 cm^2^ V^−1^ s^−1^ which is concluded as exceptionally good and was obtained due to type-1 band alignment upon heterostructure formation. The 2DEG formation at the interface is considered as major factor in improving mobility of the proposed device to due decrease in impurity scattering. The mobility delivered by proposed heterostructure FET device is reasonably recommended in many practical and potential applications.

## Data Availability

The data that supports the findings of this study are available from the corresponding author upon reasonable request.
